# MUC1 expression in Sjogren’s syndrome, KCS, and control subjects

**Published:** 2010-08-24

**Authors:** Barbary Caffery, Miriam L. Heynen, Elizabeth Joyce, Lyndon Jones, Robert Ritter, Michelle Senchyna

**Affiliations:** 1 Center for Contact Lens Research, School of Optometry, University of Waterloo, Ontario, Canada; 2 Alcon Research Ltd, Fort Worth, TX

## Abstract

**Purpose:**

To quantify and compare human mucin 1 (MUC1) protein and mRNA expression in tears and conjunctival epithelial cells collected from Sjogren’s syndrome (SS), non-Sjogren’s keratoconjunctivitus sicca (KCS) and non-dry eyed (NDE) control subjects.

**Methods:**

Seventy-six subjects were recruited for this study: 25 SS (confirmed via American-European Consensus Criteria 2002), 25 KCS (confirmed by symptoms and Schirmer scores ≤10 mm) and 26 NDE. Tears were collected using an eye-wash technique. Impression cytology was used to gather protein and mRNA from conjunctival epithelial cells. Soluble and membrane bound MUC1 were quantified via western blotting and *MUC1* mRNA was quantified by real time qPCR.

**Results:**

The SS group demonstrated significantly higher concentrations of soluble MUC1 (0.12±0.11 [SS]; 0.013±0.02 [KCS; p=0.001]; 0.0023±0.0024 [NDE; p<0.001]) and *MUC1* mRNA (3.18±1.44 [SS]; 1.79±1.18 [KCS; p<0.05]; 1.60±0.74 [NDE; p<0.05]) compared to both KCS and NDE groups. Soluble MUC1 expression was also higher in the KCS group compared to the NDE group (p=0.02), where as *MUC1* mRNA expression was similar in both KCS and NDE groups. Membrane bound MUC1 expression differed only between the SS and NDE groups (0.005±-0.003 [SS]; 0.003±0.002 [NDE; p=0.002]).

**Conclusions:**

These results demonstrate that SS subjects express greater quantities of MUC1 protein and mRNA compared to both KCS and control subjects. Increased soluble MUC1 expression was also found in KCS subjects compared to controls. Membrane bound MUC1 was present in higher concentration in SS versus NDE only. These significant changes in MUC1 expression may represent compensatory or protective responses to chronic insult to the ocular surface.

## Introduction

Mucins constitute an important part of the preocular tear film and ocular surface. It is believed that a large portion of the tear film is a mucin/aqueous gel that decreases in density from the epithelial surface toward the lipid layer [1,2] Mucins participate in the retention of water and other tear fluid components on the ocular surface and thus contribute to lubricity and healthy epithelia [3,4]. Our earliest information about ocular mucins came from the study of goblet cells [5]. However, numerous studies have now shown that multiple species of mucins, including transmembrane mucins, are derived from the ocular surface epithelium [6-10].

Mucins are present on apical surfaces of all healthy wet surface epithelia of the human body and on the glands of simple secretory epithelial tissue [11,12]. Based on the presence of common structures within their amino acid sequences, human mucins have been grouped together into two main groups: secreted and transmembrane mucins [6,13-20].

On the ocular surface, the large secreted gel-forming mucin, human mucin 5AC (MUC5AC), is expressed by the goblet cells and hydrates in the tear film to act as a lubricating agent and clearing molecule [21,22]. The membrane-tethered mucins, the most studied of which are human mucin 1 (MUC1), human mucin 4 (MUC4), and human mucin 16 (MUC16), form the dense glycocalyx at the epithelial cell-tear film interface [22] and serve to signal the cells to which they are attached [23] and to provide protection [24]. MUC16 has specifically been described as protecting the epithelium from the penetration of clinical dyes such as rose bengal [25]. There are also alternative soluble forms [25-29] of MUC1 [27] and MUC16 [26,30] found in the tear film. These alternative forms lack the cytoplasmic tail portion of the protein and are constituitively released into the tear film [11]. The functions of these soluble portions of MUC1 and MUC16 are unknown.

Mucins have been implicated in the pathophysiology of dry eye disease, but mucin deficiency is no longer described as a separate class of dry eye [31]. According to the 2007 DEWS report, dry eye disease is now classified as aqueous-deficient (AD) or evaporative (E) and within the AD dry eye group, two major subclasses exist, Sjogren’s Syndrome (SS) Dry Eye and Non-SS Dry Eye [31]. Dry eye research has shown that the gel forming mucin MUC5AC, is a major factor in dry eye disease in that its expression is reduced in dry eye subjects [32]. However, more recent research is uncovering the role of membrane spanning mucins in dry eye [24,33,34].

It is the membrane spanning mucin MUC1 that is the subject of this paper. MUC1 is the first human mucin to be cloned [35]. Its functions have been extensively studied because of its role in breast cancer [36,37]. MUC1 is the shortest [38] of the transmembrane mucins of the ocular surface. It is believed to contribute an anti-adhesive role that has been well studied in breast cancer [39]. Perhaps this property allows the lid to rub over the surface epithelium more readily. The MUC1 cytoplasmic tail is believed to be a part of many signaling events and this may allow the ocular surface epithelium to respond to changes in the environment [40]. MUC1 is also believed to play a role in epithelial protection from pathogens such as *Pseudomonas aeruginosa* [37].

In this study of MUC1 in dry eye disease, we characterize the expression of MUC1 in Sjogren’s syndrome dry eye as compared with aqueous deficient (KCS) dry eye and non dry-eyed (NDE) controls, to gain further insight into the role that MUC1 may play in dry eye disease.

## Methods

### Study design

Prior to the start of this study, ethics approval was attained from the Office of Research Ethics at the University of Waterloo and University Health Network in Toronto and all procedures adhered to the Declaration of Helsinki. A total of 76 subjects (26 non dry-eyed controls [NDE], 25 Sjogren’s subjects [SS], and 25 non-Sjogren’s keratoconjunctivitis [KCS]) were enrolled in this study. All participants underwent a clinical evaluation visit to determine entry eligibility before their second visit during which ocular samples were collected.

The inclusion and exclusion of subjects has been described in a previous paper [30]. Briefly, all SS participants had been diagnosed with primary SS at the multidisciplinary Sjogren’s Syndrome Clinic of the Toronto Western Hospital, using the American-European consensus criteria of 2002 [41]. The KCS and NDE subjects were recruited through the SS clinic and a private practice. KCS subjects had significant symptoms of dryness for over 3 months and Schirmer scores of ≤10mm in 5 min.

All subjects were free from allergy or other ocular surface diseases with the exception of blepharitis. Subjects with blepharitis were allowed to use lid scrubs and hot soaks but were not using topical antibiotics or topical anti-inflammatory medication.

### Reagents and materials

Gradient gels (NuPAGE® Novex 3%–8% Tris-Acetate Midi Gel), NuPage® tank buffer, NuPAGE® 20× transfer buffer, and molecular weight standards (Himark™) were purchased from Invitrogen (Carlsbad, CA). The transfer unit, Genie Blotter, was purchased from Idea Scientific (Minneapolis, MN). Nitrocellulose membrane and blotting paper were purchased from BioRad Laboratories (Mississauga, ON). NAP-Blocker was purchased from G Biosciences (Maryland Heights, MO). ECL-Plus™ kits were purchased from GE biosciences (Baie d’Urfe, QC). DC Protein Assay Kit ® and nitrocellulose membranes were purchased from BioRad Laboratories. Monoclonal mouse anti-human MUC1 antibody (DF3) was purchased from Signet (#61401; Dedham, MA) and goat anti-mouse IgG-HRP from Santa Cruz Biotechnology Inc. (Santa Cruz, CA). MUC1 standard antigen (CA15–3) was purchased from Calbiochem (San Diego, CA) Millipore™ Membrane Filters were purchased from Millipore™ (Fisher Scientific, Ottawa, ON). All other chemicals were purchased from Sigma-Aldrich (Oakville, ON).

### Eye wash tear collection

The methods described below have been described in detail in our previous paper [30]. Tears were collected using an eye wash method based on a previously described technique [32]. Briefly, 60 µl of sterile, physiologic saline (0.9% NaCl; Minims, Chauvin Pharmaceuticals Ltd, Romford, Essex, UK) was applied to the superior bulbar region of the unanesthetized right eye, followed by the left, and tears were collected from the inferior fornix using the micropipette. The eye washes were pooled, vortexed briefly, and placed on dry ice until transfer to −80 °C for storage.

### Conjunctival impression cytology (CIC)

Conjunctival epithelial cells were collected via impression cytology from each eye using sterile Millipore, MF membranes, (pore size 0.45 μM) [30]. Briefly, the right eye was anesthetized with two drops of a topical anesthetic (Alcaine®; Alcon, Fort Worth, TX). CIC was taken superiorly and temporally and the membranes placed in 1 ml of RLT® RNA Isolation Buffer (Qiagen, Mississauga, ON) containing 0.01% β-mercaptoethanol. The same procedures were repeated on the left eye and those two filter papers were placed in an empty sterile tube, for subsequent protein extraction. All samples were immediately placed on dry ice, then transferred to −80 °C for storage until processing.

### Protein isolation from CIC Samples

Left eye impression cytology samples were used to isolate total protein [30]. Briefly, filter papers were minced, then proteins extracted twice in a total of 75 μl extraction solution (2% sodium dodecyl sulfate; 1× Complete™ protease inhibitor cocktail [Roche, Mannheim, Germany]) at 95 °C for 10 min. Aloquoted samples were stored at −80 °C until further use.

### Determination of total protein concentration in tear and CIC samples

All total protein determinations were conducted using the DC Protein Assay Kit® following manufacture’s instructions. Each of eye wash or impression cytology extract (5 µl) was added to 5 µl of Milli-Q water and the final 10 µl was divided equally between two microplate wells to allow assay in duplicate.

### Electrophoresis and immunoblotting

Samples were thawed at room temperature and diluted to a final protein concentration of 1 µg/μl with sample buffer (247 mM Tris-HCl, pH 8.6, 2% SDS [w/v], 50 mM DTT, 1× Complete™ Protease Inhibitor [Roche], 10% glycerol, 0.002% [w/v] Bromophenol blue). Samples were heated at 100 °C for 3 min before gel loading. A titration of MUC1 standard antigen CA15–3 was run on each gel to normalize data and facilitate semi-quantitation of samples, through linear regression analysis. Following separation, protein was electrophoretically transferred to nitrocellulose membranes in a 1/10 dilution of NuPAGE Transfer Buffer. Membranes were fixed by heating at 70 °C for 30 min, air dried for 12 h then blocked in PBS + 0.05% Tween-20 (=PBS-T) + 0.1% BSA + 10% NAP for 1 h at room temperature. Blots were incubated overnight in mouse monoclonal antibody clone DF3, (1:40) in PBS-T and 0.1% BSA + 10% NAP at 4 °C then with secondary antibody (1:5000) in PBS-T + 0.1% BSA + 10% NAP for 1 h at room temperature. Blots were developed with ECL Plus® (BioRad) and chemiluminescent signals were captured by Storm840® Imaging (Molecular Dynamics, Baie d’Urfe, QC). The amount of MUC1 in each sample and standard were quantified by image analysis software (ImageQuant 5.1®; Molecular Dynamics). Known amounts of CA15–3 standard were used to generate standard curves and using the line-of-best-fit from the standard curve, the relative amount of mucin in the samples was interpolated from the graph. As multiple bands were generated in mucin western blots for all samples, all bands of 325 kDa and greater were used to quantify expression. Bands below 325 kDa were ignored.

### RNA isolation from CIC samples and reverse transcription

Tubes containing 1 ml of RLT® buffer (Qiagen) and two impression cytology samples were allowed to thaw at room temperature then vortexed. Membranes were removed using a 21 guage needle and samples were passed through a 21 gauge needle 10 times. Extraction of total RNA proceeded according to manufacturer’s directions (RNeasy® Minikit; Qiagen). The final isolation step was conducted with 40µl of RNase free water.

cDNA was synthesized from 8 µl of RNA sample using random hexamer primers with Superscript™ III First-Strand Synthesis System for RT–PCR (Invitrogen) according to the manufacturer’s instruction.

### Real Time-qPCR

Multiplex PCR reactions containing target (*MUC1*) and endogenous control (*GAPDH*) oligonucleotide primers were performed in the presence of gene-specific dye-labeled Taqman probes (Table 1). Briefly, 2 μl of cDNA were used for amplification in a 50 µl PCR reaction containing target and endogenous control oligonucleotide primers, control and target Taqman probes and Taqman^®^ Universal PCR Master Mix . Duplicate samples were used for analysis in a 7500 Real Time PCR System (Applied Biosystems, Carlsbad, CA). Conditions used for amplification were as described in our previous paper [30]. Normalized reporter dye fluorescence (R_n_) data was collected during the extension step at each cycle. Collected data was analyzed and fold-expression changes were calculated using the comparative method (2^-ΔΔCT^) of relative quantification by SDS software (v1.3.1; Applied Biosystems).

**Table 1:** Sequence data for gene amplification in real time RT–PCR.

**Gene**	**Forward primer**	**Reverse primer**	**Taqman probe**
*MUC1*	CTGGTCTGTGTTCTGGTTGC	CCACTGCTGGGTTTGTGTAA	6FAM-GAAAGAACTACGGGCAGCTG
*GAPDH*	GAAGGTGAAGGTCGGAGTCA	GACAAGCTTCCCGTTCTGAG	VIC-CAATGACCCCTTCATTGACC

### Data analysis

Statistical analysis was performed using Statistica Ver7.1 (StatSoft Inc., Tulsa, OK, USA) and Microsoft Excel™ XLfit© (IDBS, Surrey, UK) software. Graphs were plotted using Statistica Ver7 (StatSoft, Tulsa, OK).1. All data are reported as mean ±standard deviation. Statistical differences between groups for biomarker data were identified by using one-way ANOVA, and when necessary, Dunnett’s comparison of means and by Tukey’s test. Significance was identified at p<0.05 (α=0.05).

## Results

### Demographics and tear flow measurements

A total of 76 subjects were enrolled into this study. The demographics of these subjects are displayed in Table 2.

**Table 2:** Summary of demographic information for study groups.

**Group**	**Mean age (years)**	**Number of female subjects**	**Number of male subjects**	**Total subjects**
Control group of non dry-eyed	52.4±11.4	24	2	26
KCS	59.3±9.1	21	4	25
Sjogren’s syndrome	60±11.8*	21	4	25

The asterisk denotes significantly greater compared to control group (p=0.024).

The mean age of the SS group was found to be statistically higher than the NDE group (p=0.024), but not different from the KCS group (p>0.05). Mean Schirmer I scores from both eyes collected without anesthesia for 5 min revealed a significantly reduced (p<0.0001) tear flow in both SS (5.12±5.96 mm) and KCS subjects (7.84±7.35 mm), relative to NDE (23.83±7.85 mm). There was no difference in mean Schirmer I scores between the KCS and SS groups (p=0.19).

### Comparisons

**Figure 1:**
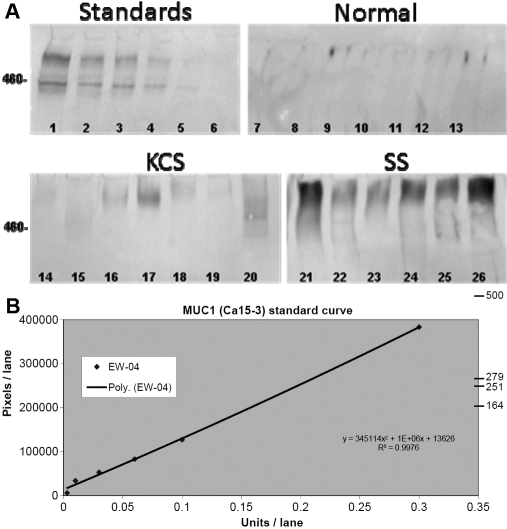
Western blot and regression analysis for soluble MUC1 quantification **A**: Examples of Soluble MUC1 western blots from tear samples derived from Normal, KCS, and SS and subjects. Lanes 1–6 are MUC1 standard antigen (CA15–3) Units; (Lane 1=0.3, Lane 2=0.1, Lane 3=0.06, Lane 4=0.03, Lane 5=0.01, Lane 6=0.003 U); Lanes 7–13 are examples of tear samples from Normal, Lanes 14–20 are from KCS, and Lanes 21–26 are from SS. **B**: A regression curve was created by graphing applied concentration of CA15–3 standard against the optical density of the resulting band immunoreactivity. Total MUC1 concentration was quantified by interpolation from this curve. MW standard (460) is listed on the left.

**Figure 2:**
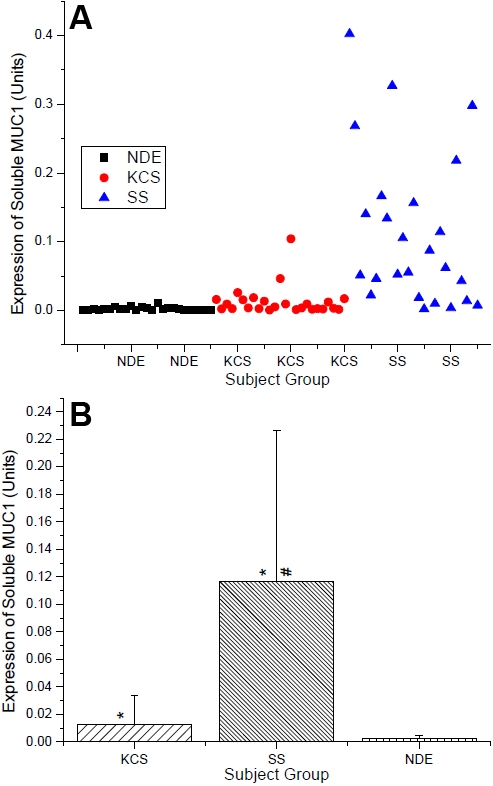
Soluble MUC1 expression as quantified by western blotting. Data expressed as (**A**) scatter graph of individual data points and (**B**) mean data. Protein samples collected via eye wash and MUC1 data expressed in Units/μg protein as calculated from interpolation from a standard curve titration of CA15–3. The asterisk indicates significantly different compared to NDE Group. The sharp (hash mark) indicates significantly different compared to KCS Group.

**Figure 3:**
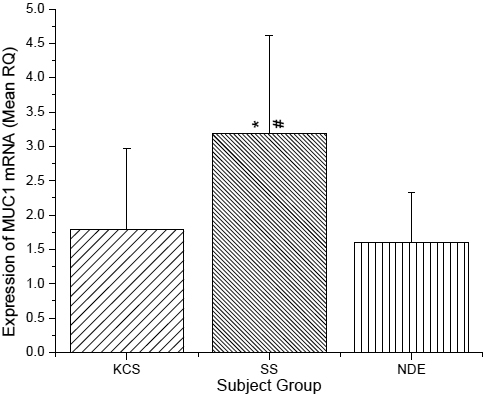
Expression of *MUC1* mRNA in conjunctival epithelial cells. RNA isolated from conjunctival epithelial cells collected via impression cytology. The asterisk indicates significantly different compared to the NDE Group and the sharp (hash mark) indicates significantly different compared to the KCS Group.

**Figure 4:**
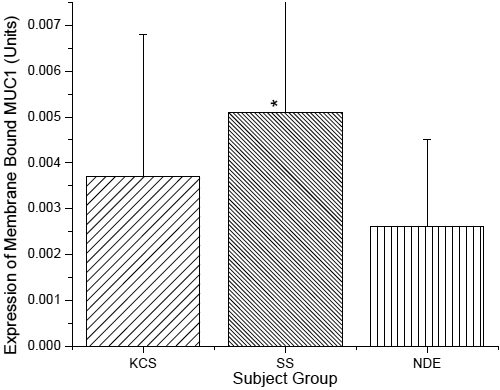
Membrane bound MUC1 expression as quantified by western blotting. Protein samples collected via impression cytology. MUC1 data expressed in Units/μg protein as calculated from interpolation from a standard curve made from titration of CA15–3. The asterisk indicates significantly different compared to the NDE Group.

Two of the 25 SS subjects did not supply sufficient tear samples for analysis of soluble MUC1, limiting this analysis to 23 of the 25 SS subjects. The SS group expressed a significantly higher concentration of soluble MUC1 compared to both KCS (0.14±0.15 [SS]; 0.013±0.02 [KCS]; p=0.001) and NDE (0.0023±0.0024; p<0.001) groups (Figure 1 and Figure 2). The SS group also expressed a significantly higher concentration of *MUC1* mRNA compared to both KCS (3.18±1.44 [SS]; 1.79±1.18 [KCS]; p<0.05) and NDE (1.60±0.75 [NDE]; p<0.05) groups (Figure 3). The KCS group expressed a significantly greater concentration of soluble MUC1 compared to the NDE group (p=0.02). Lastly, the only significant difference found in membrane bound MUC1 expression was between the SS and NDE groups (0.005±0.003 [SS]; 0.003±0.002 [NDE]; p=0.002; Figure 4), with the SS group demonstrating more membrane MUC1 than the NDE group.

## Discussion

The results of this study demonstrate that subjects with confirmed Sjogren’s syndrome have increased levels of soluble MUC1 in their tear film and *MUC1* mRNA in their conjunctival epithelial cells compared to both KCS and non dry eye (NDE) subjects and that SS subjects have increased levels of membrane bound MUC1 compared to NDE subjects. KCS subjects demonstrated increased levels of soluble MUC1 compared to NDE. To our knowledge, this is the first time that soluble MUC1 concentrations have been compared in these groups.

We note that age may have been a factor in our results, as the mean age of the two dry-eyed populations was greater compared to the control group, with significance reached in the SS group. It is known that tear volume, production, stability and /or quality is reduced in the older population [42,43], but age factors have not been determined for the status of mucin on the ocular surface. We feel that our results are relevant however, as in our study there was no significant age difference between the KCS and SS groups, while there were significant findings in mucin expression.

The authors are aware that dry eye disease is generally believed to be a disease of reduced mucin secretions. This has been demonstrated most consistently with the findings of reduced secreted MUC5AC in the tears of Sjogren’s syndrome patients [32], and in their conjunctival cells [32,44] compared to normals. KCS subjects also showed reduced MUC5AC in tears and conjunctival cells compared to normals in 3 studies [32,44,45]. One study by Zhao et al. [46] found no difference in MUC5AC levels in the tears of KCS subjects versus normals.

Very little has been written comparing transmembrane mucins in these groups. Danjo et al. [33], using histochemical staining of surface conjunctival epithelial cells gathered from impression cytology, noted reduced expression of MUC16 in non-SS dry eye and observed that this was associated with rose bengal staining. Data on other membrane bound mucins has suggested that the expression of mucosal epithelial membrane mucin (as detected by an uncharacterized antibody referred to as AMEM2), is reduced in SS and non-SS dry eyed subjects compared to normals [47].

These observations stand in contrast to our results that demonstrated no difference in membrane bound MUC1 in the conjunctiva of SS subjects compared to aqueous deficient dry eye and increased expression compared to normal subjects [30]. Our methods are certainly different as they involved cells from one eye that were pooled after collection from both temporal (exposed) and superior bulbar conjunctiva as opposed to temporal cells that were analyzed by Danjo et al. [33]. Also our testing was completed by western blot analysis. It is possible that the vagaries of glycosylation of these mucins in dry eye disease would hinder their detection using histochemical staining techniques. This inability to detect membrane bound MUC1 because of glycosylation differences is documented in the literature where the presence of MUC1 on hematopoietic cells in patients having certain cancers was ignored for some time [27].

An association between reduced membrane bound mucins, specifically MUC16, and the presence of rose bengal staining has been suggested by Danjo et al. [33] in human cells, and Argueso et al. [25] in human corneal-limbal epithelial cells lines (HCLE). If all transmembrane mucins are involved in resisting rose bengal penetration, then these data conflict in principle with our findings of increased levels of membrane bound MUC1 in SS versus NDE and no difference in membrane bound MUC16 in our previous work with SS, KCS and NDE [30], since rose bengal staining is found clinically in greater amounts in Sjogren’s syndrome patients [48]. Recent work by Argueso et al. [49], however, suggests that, in vitro, it is the interaction of the carbohydrate binding protein galectin-3 with the carbohydrate portion of both MUC1 and MUC16 that creates a protective barrier to the penetration of dye such as rose bengal. Therefore, the concentration of membrane bound mucins may not be the issue but rather their ability to interact with galectin-3.

The concentration of mRNA of transmembrane mucins in the conjunctival epithelium has been studied by others, albeit rarely. At the genetic level, a specific splice-variant of the MUC1 gene was found to be slightly reduced in dry eyed subjects [50]. In contrast, other authors have failed to find differences in MUC1 or MUC4 gene expression between controls and SS subjects [32]. To our knowledge, this is the first time that mRNA levels in human conjunctival epithelial cells have been compared in these three groups.

These results, combined with our previous work with MUC16 [30], demonstrate an excess of both MUC1 and MUC16 in the tear film of SS subjects and increased levels of mRNA for these mucins in conjunctival cells. The finding of increased tear mucins in SS is of particular interest to the clinical author (B.C.) of this study as the observation of excess mucus in the tears of SS patients is a long standing clinical finding in her experience. Mechanistically, albeit simplistically, it appears that increased expression of mRNAs coding for MUC1 and MUC16 are followed by excess shedding of these species into the tear film, or vice versa. The excess expression of mucins in disease states is a well studied phenomenon. In fact, excess mucin production in humans is an ancient defense mechanism [51] and non-ocular mucous membranes demonstrate excess mucus production under adverse conditions in dogs, rats, and humans [52-54]. Excess mucin of the ocular surface occurs most commonly in ocular allergy and here there is excess of transmembrane MUC1, MUC4, and MUC16 [55]. In vitro, inflammatory mediators that have been found in the tear film of dry eye patients have been shown to increase MUC1 expression in a human limbal corneal epithelial cell line [23]. Also, ocular cicatricial pemphigoid (OCP) patients demonstrate increased mucin production [56]. This increased mucin activity in the early stages of the keratinisation process, or during inflammation, suggests that ocular surface cells can participate in compensatory attempts to synthesize more mucin to maintain a wet surface phenotype or to protect themselves in other ways.

The shedding of the extra cellular portion of MUC1 has been studied in infection and is considered to be a reaction of the molecule to changes in its environment that then signals the cell to which it is attached [37]. It is suggested that the release of the extracellular portion produces “activation, proliferation or apoptotic response” of the epithelium [37]. Perhaps this exposed portion of MUC1 is shed because of physiologic changes associated with severe dry eye disease and this activity then signals the epithelial cells to produce more MUC1. Our findings would suggest that this compensatory or protective mechanism is most prevalent in SS dry eye disease, but may be related to disease severity, as suggested by the increased soluble MUC1 seen in KCS subjects over NDE subjects in our results. To probe this hypothesis, additional work is required with severe non-SS dry eye patients.

In summary, we quantified the expressions of ocular surface MUC1 in Sjogren’s subjects and compared them with non-Sjogren’s dry-eyed subjects and non-dry eyed controls. We found that Sjogren’s subjects express significantly elevated concentrations of both soluble MUC1 and *MUC1* mRNA compared to both KCS and NDE groups. The KCS group also demonstrated increased concentrations of soluble MUC1 in the tear film compared with NDE subjects. We propose that the conjunctival epithelium of Sjogren’s subjects reacts to dry eye changes in the tear film that cause increased shedding of MUC1 and increased production of this molecule in an attempt to protect the ocular surface and to maintain a healthy surface phenotype.
